# Editorial: Advances in understanding the pathogenetic mechanisms of neurodevelopmental disorders and neurodegenerative disease — The environment as a putative risk factor

**DOI:** 10.3389/fneur.2023.1259772

**Published:** 2023-08-03

**Authors:** Anna Maria Lavezzi, Marco Colizzi, Pamela J. Lein

**Affiliations:** ^1^“Lino Rossi” Research Center for the Study and Prevention of Unexpected Perinatal Death and SIDS, Department of Biomedical, Surgical and Dental Sciences, University of Milan, Milan, Italy; ^2^Unit of Psychiatry, Department of Medicine (DAME), University of Udine, Udine, Italy; ^3^Department of Molecular Biosciences, UC Davis School of Veterinary Medicine, University of California, Davis, Davis, CA, United States

**Keywords:** neurodevelopmental disorders, neurodegenerative disease, environmental factors, Parkinson's disease, Alzheimer's disease, sleep disorders

## Introduction

Neurodevelopmental disorders are generally influenced by not only genetic, but also intrauterine and extrauterine factors that affect the fetal-maternal environment and/or brain development that continues after birth (1). Specific genetic polymorphisms may increase susceptibility to environmental factors that alter the trajectory of brain development via diverse molecular mechanisms (2). In particular, pre- and post-natal exposure to neurotoxic metals, pesticides, persistent organic pollutants, and other chemicals is increasingly recognized as involved in the pathogenesis of neurodevelopmental disorders, such as autism, deficiency attention/hyperactivity disorders, and even fetal and infant death, including SIUDS (Sudden Unexplained Intrauterine Death Syndrome) and SIDS (Sudden Infant Death Syndrome) (3–9).

Similar evidence has been found also for neurodegenerative disorders such as Parkinson's, Alzheimer's disease, and chronic multiple sclerosis (10–12). In fact, especially in the context of specific genetic vulnerability, exposure to such environmental factors across the lifespan increases the likelihood of neurodegenerative processes. Combining research outputs from studies of both neurodevelopment and neurodegeneration may help advance our understanding of the complex phenomena that modulate brain structure and function throughout life, with implications for health and disease.

Thus, it is essential to study the ethio-pathogenetic and anatomo-pathological aspects of neurodevelopmental disorders and neurodegenerative disease with particular attention to the study of specific biomarkers useful for diagnostic and prognostic purposes.

The goal of the Research Topic “*Advances in understanding the pathogenetic mechanisms of neurodevelopmental disorders and neurodegenerative disease — The environment as a putative risk factor”* was to collect contributions from expert authors in order to advance the state of knowledge regarding the pathogenetic mechanisms by which various factors, including drugs, diet, genetic, and environmental factors, interact to increase individual risk for neurological disorders and diseases across the lifespan.

This Research Topic was proposed in particular to focus on the following subtopics:

- Genetic background that may increase susceptibility to environmental factors that alter nervous system development
- Molecular and neuropathological features of neurological disease across the lifespan
- Approaches for identifying specific genetic substrates and environmental factors that alter brain development to cause disease
- Mechanisms by which genetic and environmental factors interact to increase risk of neurological disease
- Neuropathology of unexplained perinatal deaths, considering also the interaction between environmental risk factors and brain developmental defects
- Proposal of evidence-based prevention and management strategies to decrease the incidence of these pathologies.

## Published articles

In order to summarize the contributions to this Research Topic, we have grouped the articles in two main sections: one dedicated to new perspectives useful both for preventive and diagnostic purposes of various neurological disorders and a second focused on new biomarkers which can help in predicting Alzheimer's and Parkinson's diseases.

### Section 1- Original perspectives on pathogenesis of sleep disorders, cognitive alterations and neurodegenerative processes

The articles are distributed in the following subsections:

(a) New indicators of neurodegenerative processes

- Through a meta-analysis approach, Zhang L. et al. highlighted a positive association between exposure to first-generation antiepileptic drugs (such as valproate, carbamazepine, and clonazepam) and increased risk of dementia.
- Li W. et al., in an original study found that dietary inflammation and blood inflammation indexes are negatively associated with cognitive function in an elderly American population.
- The experimental research of Li G.-S. et al. explored the ultrastructural pathological changes of the neurovascular unit (NVU), a structural and functional complex that plays an important role in the coupled interaction between neural activity and microcirculation, and consequently in the pathophysiological mechanism of many cerebral disorders. The study disclosed the presence of NVU destruction in the development stage of the cervical spondylotic myelopathy (CSM), the most common cervical spinal cord disorder among the elderly population.

(b) Involvement of environmental factors in neurological disorders

- Calderón-Garcidueñas et al. envisaged how exposures to high concentrations of particulate matter (PM2.5), ultrafine particulate and industrial nanoparticles, can damage the nervous system dating back to in prenatal life, playing a significant role in the development of neurodegenerative processes and sleep disorders.
- The adverse impact of exposure to neurotoxic metal mixtures on brain connectivity was illustrated by Invernizzi et al. through neuroimaging studies on a wide set of young people. They demonstrate that various metal mixtures may alter the brain development by modifying the global and local connectivity. These changes may potentially lead to alterations in cognition and neurobehavior in adolescents and young adults.
- Damage from toxic metals was also demonstrated by Vegard et al.. The aim of this study was to investigate whether early-life exposure to toxic metals and essential elements can adversely affect nervous system development. These authors demonstrated an association between second trimester maternal blood levels of copper and manganese and increased risk of Cerebral Palsy, which is the most common motor disability in childhood, the causes of which are currently only partly known.
- The study of Humphreys and Valdés Hernández, based on a systematic review and meta-analysis, supports the hypothesis that prenatal polycyclic aromatic hydrocarbon exposure negatively affects cognitive function and increases the risk of neurodegeneration in humans.
- Spencer et al. proposed an intensive analysis of published geographic clusters of conjugal cases, single-affected twins, and young-onset cases of Amyotrophic Lateral Sclerosis (ALS), to identify which environmental factors may trigger motor neuron disease. The study highlights that exposure to naturally occurring or synthetic hydrazine-related chemicals, acting alone or in the presence of a genetic susceptibility, is associated with the development of clinical ALS.

### Section 2- New diagnostic parameters for the early identification of individuals at risk of Parkinson's and Alzheimer's diseases

- New biomarkers for predicting the occurrence of Parkinson's disease (PD) have been highlighted by Zhang P. et al. They used bioinformatics analysis of the immune system to show that the analysis of four immune infiltration-related genes (precisely SYT1, NEFM, GAP43, and GRIA1) identified individuals at risk of PD before the onset of motor symptoms.
- The relation between hypertension and increased risk of AD has been underlined by Sáiz-Vazquez et al. in original research based on the analysis of information predominantly obtained by meta-analyses of primary studies worldwide.
- In an explorative study, Tsai et al. investigated the associations between sleep parameters measured using polysomnography and plasma levels of selected biomarkers of neurodegenerative diseases in patients with suspected obstructive sleep apnea (OSA) to assess the relationships between sleep disorders and the risk of Alzheimer's disease development. The results reveal that individuals at high-risk have significantly higher mean values for various indices of sleep-disordered breathing and arousal responses than those at low-risk.

## Conclusions

We believe that the above contributions, although heterogeneous in their approach, collectively broaden the current knowledge on the pathogenetic mechanisms of neurodevelopmental disorders and neurodegenerative diseases, which often involves complex interactions between genetic and non-genetic factors, including exposure to environmental contaminants. In conclusion, we hope these articles will prove useful for improving current diagnostic criteria and preventive strategies and also for providing impetus for further research in the field of this Research Topic.

## Author contributions

AL: Conceptualization, Data curation, Validation, Writing—original draft, Writing—review and editing. MC: Conceptualization, Data curation, Validation, Writing—original draft. PL: Conceptualization, Supervision, Validation, Writing—original draft, Writing—review and editing.
